# Rapid spread of complex change: a case study in inpatient palliative care

**DOI:** 10.1186/1472-6963-9-245

**Published:** 2009-12-29

**Authors:** Richard Della Penna, Helene Martel, Esther B Neuwirth, Jennifer Rice, Marta I Filipski, Jennifer Green, Jim Bellows

**Affiliations:** 1 Kaiser Permanente Aging Network, One Kaiser Plaza, 16th Floor, Oakland, CA 94612, USA; 2 Care Management Institute, Kaiser Permanente, One Kaiser Plaza, 16th Floor, Oakland, CA 94612, USA; 3 School of Public Health, University of California, 50 University Hall #7360, Berkeley, CA 94712, USA; 4 Caduceus Strategies, 1735 High Street SE, Salem, OR 97302, USA

## Abstract

**Background:**

Based on positive findings from a randomized controlled trial, Kaiser Permanente's national executive leadership group set an expectation that all Kaiser Permanente and partner hospitals would implement a consultative model of interdisciplinary, inpatient-based palliative care (IPC). Within one year, the number of IPC consultations program-wide increased almost tenfold from baseline, and the number of teams nearly doubled. We report here results from a qualitative evaluation of the IPC initiative after a year of implementation; our purpose was to understand factors supporting or impeding the rapid and consistent spread of a complex program.

**Methods:**

Quality improvement study using a case study design and qualitative analysis of in-depth semi-structured interviews with 36 national, regional, and local leaders.

**Results:**

Compelling evidence of impacts on patient satisfaction and quality of care generated 'pull' among adopters, expressed as a remarkably high degree of conviction about the value of the model. Broad leadership agreement gave rise to sponsorship and support that permeated the organization. A robust social network promoted knowledge exchange and built on an existing network with a strong interest in palliative care. Resource constraints, pre-existing programs of a different model, and ambiguous accountability for implementation impeded spread.

**Conclusions:**

A complex, hospital-based, interdisciplinary intervention in a large health care organization spread rapidly due to a synergy between organizational 'push' strategies and grassroots-level pull. The combination of push and pull may be especially important when the organizational context or the practice to be spread is complex.

## Background

Improving health care quality requires rapidly spreading successful evidence-based practices. However, it is widely known that innovations often fail to disseminate to other settings, even within the same health care organization, resulting in isolated improvements and missed opportunities.

While the science of implementation is still in its infancy, existing literature specific to health care suggests that innovations fail to spread due to factors that include: characteristics of the innovation and of individual adopters; characteristics of the dissemination and assimilation processes; patterns of organizational communication, influence and linkage; the organization's readiness for change; and the external context within which it operates [1].

Specific factors implicated in the failure of innovations to spread within health care include: ideas that lack clear practical, clinical, and cost advantages, simplicity, trialability, observability, or compatibility with current organizational practices, norms, and culture; inattention to social aspects of change and individual readiness to change; ambiguous or convoluted channels of communication; poor design of change messages; tepid endorsement by opinion leaders; lack of senior leadership support; inadequate opportunities for early adopters to directly teach others; intolerance for reinvention and refining of ideas; inadequate plan for and measurement of spread; and separating spread of innovations from continuous quality improvement activities [2-7].

Accelerating effective, sustainable dissemination of improvements in health care's complex adaptive systems is a pressing issue [8]. A variety of frameworks to facilitate this have been suggested [9]. However, few examples exist in the literature of successful spread of complex interventions in large organizations. We report here on an initiative to disseminate a complex inpatient palliative care program within a large integrated delivery system in the US, shedding light on how one initiative overcame barriers to spread.

### The Inpatient Palliative Care Initiative

In 2005, Kaiser Permanente completed a multi-center randomized controlled study of three distinct palliative care models. The study followed nearly a decade of innovation in end-of-life care in home, office, and inpatient settings, and strong interest existed across Kaiser Permanente regions to identify best practices.

The most successful model demonstrated favorable impacts on patient satisfaction and clinical outcomes. The cornerstone of the inpatient palliative care (IPC) model is an interdisciplinary team functioning collaboratively with patients, families, and hospital staff (Table 1). Its central goals are to help patients identify and communicate their values and health care preferences as they near the end of life and to align future care with their preferences; the IPC team does not provide direct care, which remains the responsibility of each patient's existing care team. Many patients do not prefer intensive treatment with little hope of success [10], so aligning care with patient and family preferences can improve the care experience for patients and families and decrease average costs after hospital discharge [11,12].

**Table 1:** Features of the IPC model

**Interdisciplinary team**	Consists of physician, nurse, social worker, and chaplain Consults with patient and family as a team for 1-2 hours in the hospital to identify the entire range of issues: medical, social, emotional, and spiritual Available on site Monday through Friday, by phone weekends and evenings for patient, family, and other clinical staff
**Consultative approach**	Team consults with patient, family, and attending physician Team does not assume care for patient; team supports hospitalist and other clinical staff treating the patient

In May 2006, Kaiser Permanente's national executive leadership group reviewed the findings of the randomized controlled trial and the projected outcomes of implementing the IPC model across all Kaiser Permanente regions. At the time, regional authorities made independent decisions about program development. Medical centers in four of eight regions had some form of palliative care; however, only one used the interdisciplinary team-based consultative model. The projection suggested the potential for significant improvements across the entire organization in quality, patient satisfaction, and cost outcomes that aligned closely with leadership priorities. National leadership consequently set an expectation that all Kaiser Permanente and partner hospitals would implement the IPC model, beginning in January 2007.

### The plan for spread

Support for IPC quickly diffused throughout the organization. A national council of regional operations leaders promoted the initiative at the regional level. National palliative care leaders formed a core team to support the regions as they implemented the model. The members of this team included national and regional clinicians with expertise and interest in palliative care and experts in spread and implementation, training, evaluation, communications, and measurement. Working closely with other national and regional clinical leaders, the national IPC team identified implementation leads in each region responsible for getting local teams up and running.

The initiative started with clear specification of the model, namely, an interdisciplinary team functioning collaboratively to consult with patients, their families, and care providers to align care with preferences without assuming responsibility for care. Specific activities designed and implemented to support dissemination of the IPC model included a national kick-off meeting that presented the model and the evidence supporting it and created informal networks, trainings for regional IPC team members and program managers, sponsoring visits by new teams to the originating team, and enhancing an existing network for end-of-life issues.

The national team provided adopting sites with standard tools that could be adapted to local conditions. Developed from tools at the originating site, they included a business case and an operations manual detailing team structure, roles, and processes. Online training materials were developed to augment live training activities. A dedicated web site facilitated knowledge exchange and networking. Videos and brochures about palliative care were developed to provide consistent messaging across Kaiser Permanente. Outreach to hospital attending physicians encouraged them to refer eligible patients. The national team identified a data collection set--a 'dashboard'--for tracking activity and performance across regions.

The spread initiative built on an existing robust internal network focused on end-of-life issues. The national IPC team increased networking opportunities with interregional teleconferences and held monthly problem-solving meetings with local IPC teams. Leadership at the originating site made their team available for new teams to visit and directly observe consultations and team functioning.

One ingredient the national initiative did not provide was funding for local staffing and operations. Each adopting site was asked to identify resources from within existing budgets; this was typically a combination of funds from hospital, health plan, and medical group budgets.

The initiative represented an organization-wide, concerted movement that occurred within the context of ongoing quality improvement activities in palliative care at the regional and local levels. It followed years of rapid cycle quality improvement activities, and iterative methods were used to refine local implementation of the IPC model.

### Extent and pace of spread

**Figure 1:**
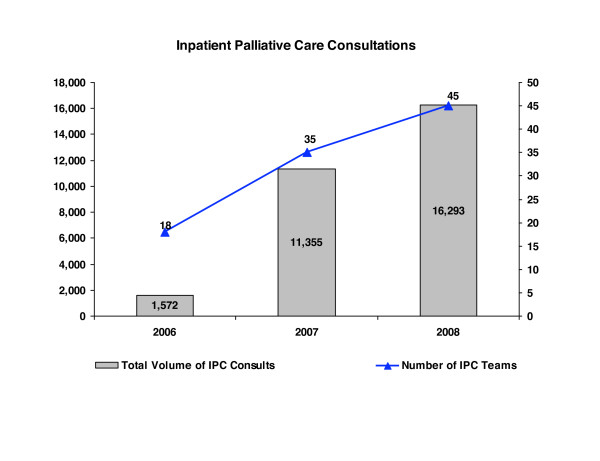
**Spread of IPC model**. Increase in inpatient palliative care consultations over time.

Seven of Kaiser Permanente's eight regions, accounting for more than 98% of members, began implementing IPC teams in January 2007. Within one year, the number of IPC consultations programwide increased almost tenfold from the baseline established by pre-existing palliative care programs, and the number of teams nearly doubled. In 2008, 45 teams were in place and consultations continued to rise. Within twelve months, the recommended team-based model had moved from a single demonstration site to all 32 Kaiser Permanente hospitals and five partner hospitals in participating regions (Figure 1). We report here results from a qualitative evaluation of the IPC initiative after a year of implementation; our purpose was to understand factors supporting or impeding the rapid and consistent spread of a complex program.

## Methods

### Setting

Kaiser Permanente is the United States' largest private, not-for-profit integrated health care delivery system with more than 8.5 million members. It addresses all health care needs for its members, from primary and preventive services to hospital care.

Kaiser Permanente has more than 13,000 physicians and 156,000 technical, administrative, and clerical personnel in eight geographic regions. It operates 32 hospitals nationwide and also contracts with others to provide inpatient care to members. In each region, physicians join partnerships or professional corporations contracting exclusively with the not-for-profit Kaiser Foundation Health Plan and Kaiser Foundation Hospitals, assuming full responsibility for members' medical care. Each Kaiser Permanente region is separately governed and managed by a partnership consisting of the executive director of the regional physician-owned medical group and the president of the regional health plan and hospitals entities.

### Data collection and analysis

For this quality improvement project, we used a case study design to understand factors promoting and inhibiting the spread of the IPC program. Consistent with validated theoretical sampling methods for qualitative research, participants and sites were chosen purposefully to maximize variation on the key dimensions of interest [13,14]. We interviewed a range of national and regional leaders from Kaiser Permanente's health plan, hospitals, and medical groups, using purposive and convenience sampling to identify individuals from a list generated by key informants. From each region, we selected at least one senior leader, one implementation leader, and one member of a team implementing IPC, oversampling in large regions. Senior leaders had broad responsibility for many aspects of patient care, and implementation leaders focused on palliative care or elder care. We also selected several members of the national IPC team and senior national Kaiser Permanente leaders. In all, we conducted 36 in-depth semi-structured interviews by telephone or in person.

The evaluation was designed for quality improvement purposes; its primary functions were to inform refinements to the continuing spread effort and to inform future organization-wide spread efforts. Interview questions were based on existing literature on spreading innovations in health care and tailored to meet organizational needs (Table 2). Selected stakeholders reviewed a list of key domains for interview questions and suggested edits and additions.

**Table 2:** Summary of Interview Instruments

Main Inquiry Domains	Questions	Examples of probe topics
**Respondent's role**	**What has been your role in spreading IPC?**	Involvement in earlier palliative care research Role in the organization; e.g., leadership level

**Choice of IPC model**	**Why do you think this intervention was chosen for organization-wide spread?** **When choosing a model to spread, was the selection of palliative care one of your top three priorities?**	Perceived problem that needed a solution Value of the model Priority for choosing a palliative care model Motivation for choosing model from randomized controlled trial

**Sponsorship and** **organizational support**	**Can you explain your understanding of how the IPC model was sponsored and how sponsorship may have changed over time?** **How did the organization-wide support work for you and your teams?**	Relationships formed Seed money How was the model received? How important was the evidence base? How important was exact replication? How important has local sponsorship been to successful IPC implementation? How important has national sponsorship been to successful IPC implementation?

**Organization-wide initiative**	**What are the advantages and disadvantages of spreading an initiative like IPC across the organization?**	Value of being part of an organization-wide spread effort Challenges Local response

**Implementation and dissemination**	**How has implementation and dissemination of the IPC model gone in your area [over time]?** **How has implementation and dissemination of the IPC model gone in the different areas [over time]?**	What worked? What didn't? What could be improved? Readiness to change Previous palliative care experience/programs Key implementation roles Change management strategies Facilitators and barriers to spread Local benefits of IPC model Resources needed Ongoing program assessment activities Usefulness of organization-wide strategies (trainings, communication tools, webinar, outcome measures, Operations Manual) How successful do you think the spread of IPC has been so far? How did your experience of spread of IPC compare with other spread experiences?

**Lessons learned**	**What lessons have you learned about being part of spread of an organization-wide model?**	Lessons learned to help support other organization-wide spread initiatives Recommendations based on your experience

Interviewee identities were confidential, and data were stripped of identifiers. Interviews were recorded and transcribed. ATLAS.ti software, a qualitative data management tool, facilitated analysis; we used standard qualitative methods for iterative theme identification and coding [15]. We combined inductive and deductive methods, drawing from grounded theory, analytic inductive methods, and constant comparison [16-18]. Three authors reviewed several initial interviews, comparing them to the list of key domains and noting where domains were useful in interpreting interview data and where previously unidentified themes arose. A final set of codes was developed to analyze the full set of data. They encompassed leadership priorities, communication and action; barriers to spread; facilitators of spread; status of implementation; consistency with the IPC model; usefulness of data and analytics; and recommendations and lessons learned. Three authors coded all 36 interviews, which were then analyzed iteratively for common themes.

## Results

### Factors promoting spread

The evidence behind the IPC model, demonstrated by the randomized trial, was an important factor promoting its spread. Demonstrated impacts on care quality and patient satisfaction were compelling, and the potential impact on costs prompted one interviewee to call it a "win-win-win" innovation. As a member of a new IPC team explained,

"It was definitely evidence-based in that the IPC model far outweighed other models in providing what the research indicates people want." (Implementation leader #1)

A senior leader put it this way:

"First and foremost, members realized this was great care. We need to do this not only as great quality care, but doing the right thing is also saving our members money." (Senior leader #2)

Leadership support and sponsorship were pivotal at all organizational levels. National leadership provided a clear message about the importance of implementing IPC. As one implementation leader noted,

"When you have leadership at the highest levels acting as sponsors, you take the work really seriously." (Implementation leader #3)

The national council of regional operations leaders endorsed the spread initiative and committed to mutual accountability for implementing the consultative IPC model throughout Kaiser Permanente. Acting in their own regions, operations leaders brokered working agreements between hospital chief operating officers and physician medical group directors. These agreements included funding arrangements for the IPC teams. An implementation leader explained,

"I don't think it would have gotten off the ground if we hadn't had the support of the administrative team. I don't think we would have had their support if it wasn't for the operational leaders." (Implementation leader #4)

Vital sponsorship and support activities included removing obstacles, designating teams and funding staff, assigning space, communicating the IPC model's importance to the organization at large, and raising awareness among hospital staff about appropriate palliative care referrals. A member of a new team said,

"Our medical director has been a very strong supporter. He works with hospital leadership to tackle obstacles... things as small as having a room for family conferences." (Implementation leader #5)

Systems and opportunities for social networking facilitated the transfer of explicit and tacit knowledge. An interregional network of elder care champions, in existence for five years prior to the spread initiative, provided a cohesive network of clinicians dedicated to end-of-life care. As IPC implementation began, Kaiser Permanente provided logistic support for enlarging this network. As one senior leader said,

"We have regular telephone calls regarding the program. I feel a connection with the other regions and with national. It has worked very well for us, too." (Senior leader #6)

Similarly, a member of a new IPC team commented,

"...On the calls, it's nice to know we're all struggling with the same issues and the same problems..." (Implementation leader #7)

In addition, the ability to observe the IPC model at the original site was important. Interviewees frequently mentioned a pivotal experience when a new team member observing a patient consultation for the first time understood the nature or importance of IPC teams in a new way. For example, a senior leader noted,

"Site visits were critical for some of the teams. They had a huge 'aha' moment when they saw the model in operation at the original site. They thought they understood it, but then they saw it and said, 'Oh my God, we didn't get it until now."'(Senior leader #8)

Finally, many individuals expressed a remarkably high degree of conviction about the IPC model. They expressed it in comments like the following:

"I want to reiterate that this is a great program, and I'm really thankful for the benefits we know our members receive. They express that very sincerely. It's much, much needed. I hope it never goes away." (Implementation leader #1)

"The feedback we received led me to believe that many people were inspired by the trainings, truly excited about doing this work." (Implementation leader #9)

"I deeply believe that if you took time to really talk to people about their goals and preferences, many would say, 'This isn't helping me and I don't want all this stuff. I don't want to tether the rest of my life to medical care, dying in an ICU."' (Senior leader #10)

### Factors inhibiting spread

Interviewees also spoke about factors impeding IPC spread. Lack of resources was frequently mentioned. Local resource constraints sometimes necessitated a team that lacked one or more professional disciplines (i.e., physician, nurse, chaplain, social worker), rotated different providers through the IPC team, or operated less than full time. As a senior leader noted,

"It has been hard for the regions and the medical centers to find the funds and be able to prioritize." (Senior leader #8)

Another senior leader commented,

"(Resources) weren't enough to cover the model, so we had to scramble and shift things around to be as true as we could to the model." (Senior leader #11)

Funding issues were compounded by a lack of clarity about which Kaiser Permanente entity--hospitals, health plan, or medical groups--was responsible for providing resources for the new programs. As a senior leader explained:

"Different regions did different things. (One region) made some start-up regional funding available to hospitals to get a program and people in place, but they had to find the money for the next year. Another region just said, 'We want you to do this.' There's a whole series of negotiations at the hospital between the physician-in-charge and the chief operating officer about whose budget will support what." (Senior leader #12)

Another leader stated,

"All hospital directors think it's the right thing to do; it's just tough to put something like this into place. The biggest factor was getting the region to come up with at least a portion of the funding." (Senior leader #11)

Ambiguous responsibility for funding IPC carried over into unclear accountability for program implementation, which some leaders said impeded spread. As one of the leaders from the original site explained,

"We demonstrated and explained from our perspective why we thought the model was effective and why we saw the results we did. There was no accountability or testing to see if they were going to be fitting into the model." (Implementation leader #9)

While sponsorship and support from leaders were strong, they were not universal. Some interviewees noted that gaps in leadership alignment impeded implementation at their sites. As an operational leader explained,

"We lost some vital leadership over the last four to five months. And so the strong sponsorship for this hasn't been as visible as it could be." (Senior leader #2)

Previous local experience with palliative care also influenced the consistent and rapid spread of the IPC model. As a member of an implementing team stated,

"There were a variety of approaches in this region, because each program has grown opportunistically in its environment... Some very strong programs don't use the (recommended) model." (Implementation leader #13)

Sites with pre-existing palliative care programs tended to move more slowly to adopt the recommended team-based consultative model.

## Discussion

The speed and scale of spread of this complex interdisciplinary inpatient care program are noteworthy, given the well-documented difficulty of disseminating successful practices throughout large, complex organizations [19]. Our interviews indicated key factors supporting rapid spread. They included a strong evidence base, broad leadership support, and a robust social system promoting formal and tacit knowledge exchange that built on an existing network with a strong interest in palliative care. Factors impeding spread included resource constraints, unclear responsibility for funding and accountability for implementation, and pre-existing ways of providing palliative care.

Strengths of our study include the fit between the quality improvement nature of our objective and our ability to conduct in-depth interviews with a range of leaders in different positions and at different sites throughout a large integrated health care delivery system. Limitations include the fact that our results came from a single organization and have unknown applicability to other settings. In addition, we did not attempt to quantify the relative importance of factors that leaders perceived as facilitating or impeding dissemination or elucidate whether response patterns varied between leaders in different positions.

Factors reported by leaders as promoting dissemination of the IPC model overcame barriers identified in the literature and noted in the background section of this article. The randomized controlled trial provided compelling evidence that the IPC model had clear advantages in terms of outcomes, patient satisfaction, and costs. Senior leadership support was evident throughout the organization. The social system comprising the existing clinical network that focused on aging included organizational opinion leaders, made use of existing effective and straightforward communication channels, and allowed for formal and informal interactions and education that supported social and psychological dimensions of change.

Similarly, factors impeding model dissemination exemplified barriers to the spread of better ideas in health care that were also identified earlier. A climate of cost containment in the United States, coupled with multiple competing organizational priorities, meant that dedicated resources were not fully sufficient. Linkages between different parts of the system contributed to unclear responsibility for funding. Like many complex adaptive systems in health care, Kaiser Permanente is a loosely-coupled organization; leadership is decentralized, with connections of variable strength between organizational segments, i.e., national, regional and local leadership and health plan, hospitals and medical groups. The inevitable result is some degree of ambiguity about priorities [20]. Sites with pre-existing palliative care programs were invested in their existing models.

We found that the available literature on disseminating better practices in health care did not capture the sometimes intense sense of personal engagement that permeated all levels of the organization. While motivation of individuals can influence the uptake of new knowledge [21], this broad personal engagement seemed to be a force stronger than individuals' rational beliefs about the benefits of IPC. We also viewed it as distinct from "push," here represented by the planned activities undertaken to implement national leadership's expectation that the model would spread to all hospitals.

We recognized this force as "pull," a true desire on the part of many leaders and staff to implement IPC because it resonated with deeply-held personal and organizational values. The concept of pull originated in Toyota's LEAN methodology, which eliminates waste by tying production to demand arising from consumer-perceived value [22]. It has been more loosely applied within health care and refers here to change that arises as a result of collective action by inspired and mobilized people [23,24]. Pull can occur when a change proposition presents an "irresistible emotional and logical argument that fits with the values, beliefs and life experiences of the clinicians and managers it is targeted at" [23]. The strong evidence base and business case contributed the logical underpinnings to the argument for implementing the IPC model; improving care during advanced illness and dying resonated with leaders and team members because of professional and/or personal life experience. The latter is also likely to be the case in other palliative care initiatives [25-27]. In addition, the IPC initiative, like the 100,000 Lives campaign [28], offered a clear way to make a difference in the lives of patients through an identified process and allowed supporters to demonstrate their values as part of a wider movement [29].

The leaders we interviewed cautioned that the intense engagement engendered by the spread of IPC could not necessarily be recreated in subsequent initiatives. What elements (e.g., clinical area, immediacy of impact, and others) constitute a compelling emotional argument is an important area for further research.

**Figure 2:**
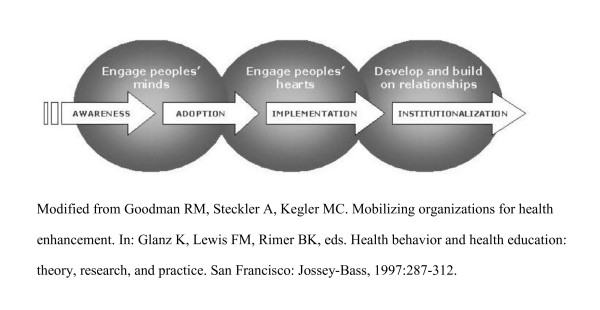
**Facilitating rapid spread of the IPC model**. A graphic depiction of the process of rapid spread.

In our case, the push of effective organizational strategies was interwoven with the pull of emotional appeal and the momentum of something akin to a social movement. The expectation of broad implementation from national leadership reverberated throughout all levels of the organization, and a variety of effective tools for rapid implementation were available. Taken together, these elements fueled the rapid spread of IPC teams (Figure 2).

We also learned about variability in spread. Migrating to a recommended model when clinicians and leaders are invested in pre-existing palliative care program poses different challenges than does implementing a model where nothing similar exists. Variability also occurred when some leaders believed that precisely replicating the tested model was relatively unimportant and that they could develop a model better suited to local conditions.

Some teams that did not replicate the IPC model accurately later found their programs were less successful than expected; over time, they moved toward greater accuracy. Iterative quality improvement processes were supported by the data dashboard developed by the national IPC team and shared with regions on a quarterly basis. Rapid-cycle quality improvement or Plan-Do-Study-Act cycles are common methods for quality improvement within Kaiser Permanente as elsewhere. They reliably produce improvements in end-of-life care when used alone or in combination with more traditional research methodologies, such as the randomized controlled trial that provided the case for the IPC model [30-32].

Important questions about the impact of the IPC initiative remain unanswered. Evaluation is in process of its impact on cost, quality, and satisfaction, with quantitative and qualitative metrics including a survey of bereaved families. An additional longitudinal follow-up ethnographic study on patient and family care experiences includes a qualitative assessment of the experiences of IPC staff and referring clinicians.

## Conclusions

Our analysis of the spread of a hospital-based, complex, interdisciplinary innovation in a large health care organization led to a greater understanding of how organizational push strategies and grassroots pull can contribute synergistically to spread a clinical practice that can significantly improve quality and patient satisfaction. Organizations that seek rapid spread of successful practices should evaluate whether an active or latent grassroots base exists that can be mobilized to generate the pull of emotional appeal and a sense of being part of a social movement, in addition to using the push of leadership alignment, standardized tools, and accountability to support dissemination. The combination of push and pull may be especially important when the organizational context or the practice to be spread is complex.

## Competing interests

The authors declare that they have no competing interests.

## Authors' contributions

RDP was clinical lead for the IPC initiative, making essential contributions to every phase of this work. HM provided essential support to the IPC teams and participated in the design of the study and data analysis. EN led the quality improvement study design, data analysis, data collection, and revised the manuscript for important intellectual content. JR was involved in all stages of the study, from conception through write-up. MF collected interview data and contributed to data analysis and manuscript revision. JB contributed to study design and data analysis and interpretation and revised the manuscript for important intellectual content. JG revised the manuscript for important intellectual content and contributed to the write-up. All authors read and approved the final manuscript.

## Pre-publication history

The pre-publication history for this paper can be accessed here:

http://www.biomedcentral.com/1472-6963/9/245/prepub
